# Social networks reveal sex- and age-patterned social structure in Butler’s gartersnakes (*Thamnophis butleri*)

**DOI:** 10.1093/beheco/arad095

**Published:** 2023-11-14

**Authors:** Morgan Skinner, Megan Hazell, Joel Jameson, Stephen C Lougheed

**Affiliations:** Department of Psychology, Wilfrid Laurier University, 75 University Ave West, Waterloo, ON N2L 3C5, Canada; Department of Biology, Queen’s University, 99 University Ave, Kingston, ON K7L 3N6, Canada; WSP, 1600 Boulevard Rene-Levesque West, 11th floor, Montreal, QC H3H 1P9, Canada; Department of Biology, Queen’s University, 99 University Ave, Kingston, ON K7L 3N6, Canada

**Keywords:** aggregation, age segregation, gartersnakes, sex segregation, social structure, social behavior

## Abstract

Sex- and age-based social structures have been well documented in animals with visible aggregations. However, very little is known about the social structures of snakes. This is most likely because snakes are often considered non-social animals and are particularly difficult to observe in the wild. Here, we show that wild Butler’s Gartersnakes have an age and sex assorted social structure similar to more commonly studied social animals. To demonstrate this, we use data from a 12-year capture-mark-recapture study to identify social interactions using social network analyses. We find that the social structures of Butler’s Gartersnakes comprise sex- and age-assorted intra-species communities with older females often central and age segregation partially due to patterns of study site use. In addition, we find that females tended to increase in sociability as they aged while the opposite occurred in males. We also present evidence that social interaction may provide fitness benefits, where snakes that were part of a social network were more likely to have improved body condition. We demonstrate that conventional capture data can reveal valuable information on social structures in cryptic species. This is particularly valuable as research has consistently demonstrated that understanding social structure is important for conservation efforts. Additionally, research on the social patterns of animals without obvious social groups provides valuable insight into the evolution of group living.

## Teaser

Gartersnakes have female-centric communities. Because snakes are secretive and cryptic, little is known about their social structure. We use capture-mark-recapture data and social network analysis to access the social structure of Butler’s Gartersnakes. We find that gartersnake social structures share characteristics with those of more commonly studied mammals and birds. Generally, gartersnakes associate with individuals of the same sex and age, and both older snakes and females are central to the resulting communities.

## INTRODUCTION

It is common for animals to aggregate in groups (Krause and Ruxton 2002). Such groups take many forms, from relatively small groups with stable membership (e.g., Greater Capybara, *Hydrochoerus hydrochaeris*; Herrera et al. 2011) to large seasonal mating aggregations (e.g., Zebra Shark, *Stegostoma fasciatum*; Dudgeon et al. 2008). Within animal groups, there are often demographic (i.e., sex and age) differences that influence social structure. For example, sometimes the oldest or largest individual will play an important role in their respective groups. This often occurs in groups in which older, dominant individuals help maintain group stability (Newberry and Cloutier 2000; Bonanni et al. 2017; Tibbetts et al. 2022) but can occur for other reasons, such as when individuals have valuable experiential information (McComb et al. 2011). When these roles result in individuals becoming a common social link between individuals or groups of individuals, they are often considered to be central to their social network (Farine and Whitehead 2015). Beyond central individuals, social roles can differ more generally based on sex and age. For example, in Vervet Monkey (*Chlorocebus pygerythrus*) groups, females participate more in grooming interactions with each other, whereas juvenile males participate more in play interactions with one another (Canteloup et al. 2021). In this case, social interactions are thought to be a result of male-biased dispersal and female philopatry; the males form play-bonds with individuals that they will disperse with at maturity, and females focus on long-term social bonds with other females (Canteloup et al. 2021). This pattern of behavior occurs in other primate species, and results in a form of sex-assorted structure with groups consisting of related adult females, their young, and immigrant adult males (Harcourt 2010; Beisner et al. 2021). Demographic-dependent sorting is a common type of group structure across taxa, and can involve individuals of the same sex or age interacting more with each other than with individuals of a different sex or age (Ruckstuhl and Neuhaus 2005). Interactions between individuals with the same or similar demographic traits is often referred to as homophily, and when looking at a population broadly, this can result in sex- and/or age-assorted community structures (Lusseau and Newman 2004).

Sex-assorted intra-species communities are particularly pronounced in some taxa. For example, in ungulates, adult males and females often live separately outside of breeding season (Ruckstuhl and Neuhaus 2000). Sex-dependent sorting can also interact with age; for some ungulates, young males will remain in predominantly female groups while older males remain separate (Bon et al. 2005). Cetaceans also demonstrate sex-assorted structures that are sometimes linked to age (Wearmouth and Sims 2008). For example, in Sperm Whales (*Physeter macrocephalus*), females remain in matrilineal communities while males disperse from these family units, form bachelor communities, and then become increasingly solitary as they age (Wearmouth and Sims 2008). Although female-centered matrifocal communities are common in these animal groups, sex and age differences in grouping patterns can lead to other forms of sex-assorted structure. For example, in some species, a community may share the same habitat but segregate by sex (González-Solís et al. 2000; Breed et al. 2006) and/or age (Wikelski and Trillmich 1994; Pelletier et al. 2014) while foraging. In Green Sea Turtles (*Chelonia mydas*), adult females form communities in female-only zones that males generally avoid (Booth and Peters 1972).

The extent to which sex-assorted social structures are the result of differences in social behavior is debated, primarily within the sex segregation literature (Main 2008; Wearmouth and Sims 2008). Males and females in such social groups often have different activity budgets and thermoregulatory needs, and are sexually dimorphic. This means that individuals have different resource requirements that could drive sex-based sorting independent of sociality (Ruckstuhl and Neuhaus 2002; Wearmouth and Sims 2008). Yet, in some animal species, there is evidence that same-sex affiliation and/or opposite-sex avoidance may be a proximate cause of segregation (Green Sea Turtles; *Chelonia mydas*; Booth and Peters 1972; Bottlenose Dolphins; *Tursiops truncatus*; Wearmouth and Sims 2008; Soay Sheep; *Ovis aries*; Pérez-Barbería et al. 2005). Some of the proposed causes of sex-assorted structures apply equally to age-sorting; however, the reasons for sex and age sorting within a species may differ. For example, in Eurasian Oystercatchers (*Haematopus ostralegus)* sex-dependent sorting is the result of differences in habitat preference, whereas age sorting occurs due to social factors (Catry et al. 2005).

These aforementioned examples illustrate that much of what we know about the role of sex and age on social structure comes from research on taxa with visible social aggregations, such as mammals and birds (e.g., Farine et al. 2015). Less is known about the grouping patterns of animals with more cryptic social interactions, such as snakes (Bonnet et al. 2002; Hatchwell 2010; Doody et al. 2013) which are often difficult to find and monitor (Durso et al. 2011). As snakes are highly secretive, and often communicate social information through invisible chemical cues (Halpern and Martínez-Marcos 2003), understanding how snakes interact is particularly challenging. Indeed, snakes are often incorrectly perceived as non-social (Brattstrom 1974) and studies of their sociality are rare (Bonnet et al. 2002; Stahlschmidt 2011). Despite this, research on snake behavior continues to demonstrate that some species share social behaviors with more commonly studied animals; this includes basic parental care (Vipers; Greene et al. 2002; Southern African Python; *Python natalensis*; Alexander 2018), dominance hierarchies (Indian Python; *Python molurus*; Barker et al. 1979), preferred associations (Arizona Black Rattlesnake; *Crotalus cerberus*; Schuett et al. 2017; Eastern Gartersnake; *Thamnophis sirtalis sirtalis*; Skinner and Miller 2020), public information use (Timber Rattlesnake; *Crotalus horridus*; Clark 2007), and territoriality (Taiwanese Kukri Snake; *Oligodon formosanus*; Huang et al. 2011). Additionally, research on snake grouping patterns has found interactions between related females similar to those found in species with matrifocal social groups. For example, female Cottonmouths (*Agkistrodon piscivorus*; Hoss et al. 2015) and Timber Rattlesnakes (Clark et al. 2012) preferentially affiliate with related females and related gravid individuals, respectively. Beyond female-focal kin groups, recent laboratory research on gartersnakes found demographic-dependent differences in sociability that could translate to age or sex sorting within communities of wild snakes (Skinner and Miller 2022; Skinner et al. 2022). In particular, research on changes in social attraction in Eastern Gartersnakes (*Thamnophis sirtalis sirtalis*) over the first 8 months of development found that female snakes became more attracted to a social stimulus derived from conspecific skin lipids as they grew, and male snakes became less attracted to a social stimulus as they grew (Skinner et al. 2022). These demographic-dependent patterns of social attraction could translate to social groups similar to those found in species in which female communities live separately from more solitary adult males.

The secretive nature of snakes has meant that studies examining their grouping patterns in the wild are rare, and evidence for social grouping is limited. In one of the few studies that examined snake group sizes outside of seasonal aggregations, there was limited evidence that group sizes were larger than what would be expected by chance (Gregory 2004). It can also be difficult to disentangle to what extent aggregations are driven by sociability, mutual attraction to resources, or both (Spiegel et al. 2016). Despite these challenges, analyses of shared space use have provided valuable information on social patterns in squamates (Spiegel et al. 2017). Turner (2023) examined Little Whip Snake (*Suta flagellum*) aggregations at common refuge sites and found evidence of non-random associations between juveniles and opposite sex pairs, as well as male–male social avoidance. Radiotelemetry studies have also inferred conspecific avoidance of male Eastern Brown Snakes (*Pseudonaja textilis*; Whitaker and Shine 2003), social constraints on the home ranges of Broad-headed Snakes (*Hoplocephalus bungaroides*; Webb and Shine 1997), and male avoidance and female attraction in Cottonmouths (*Agkistrodon piscivores*; Roth and Lutterschmidt 2011). Data on capture locations have also been used to infer social interactions. For example, Shine et al. (2005) used capture-mark-recapture data to infer long-term social bonds in Turtle-headed Sea Snakes (*Emydocephalus annulatus*).

In this paper, we examined capture location and demographic information that was collected during a 12-year capture-mark-recapture project on Butler’s Gartersnakes (*Thamnophis butleri*) in Ontario, Canada. We used this information to infer the snakes’ interaction patterns and social structure using social network analysis. To do this, we looked for effects of sex, age, and body condition on sociability and social structures. With recent laboratory research showing age-dependent sex differences in social behavior in gartersnakes (Skinner et al. 2022), we hypothesized that these differences would directly translate to a sex- and/or age-assorted social structures in wild Butler’s Gartersnakes. We predicted that social affiliation in older females, and social avoidance in older males, would lead to prevalent female-female social connections (i.e., female homophily). To further characterize our hypothesized social structure, we subdivided the gartersnake social networks into communities of associating individuals, and looked for sex and age differences in the snakes that were central to their communities. Further, we looked for relationships between body condition (i.e., physical health) and social network membership; since group living has numerous benefits (Majolo and Huang 2017), we predicted that the benefits of group integration may be reflected in the snakes’ health. As habitat variation could influence association rates, we tested if the number of observed associations differed from two null models in which a snake’s time and location of capture were broadly constrained but either the exact date or capture location was permuted. Further, we looked for sex, age, and body condition differences in study site usage—measured by capture rates. Our study provides further evidence on the complexity of snake sociality and demonstrates that a capture-mark-recapture framework in combination with social network analyses can be useful for studying sociality in cryptic species. We note that inferring social associations from temporal and physical proximity has been used as evidence for social structure in both snakes (Whitaker and Shine 2003; Shine et al. 2005) and lizards (Osterwalder et al. 2004; Spiegel et al. 2017). The novelty of our approach is the combination of social network analysis and capture-mark-recapture data.

## METHODS

### Study species

The Butler’s Gartersnake is a small to medium sized diurnal snake that inhabits areas of the northern United States and small sections of southern Ontario, Canada, where it is classified as endangered (COSEWIC 2010; COSSARO 2011). Butler’s Gartersnakes display sexual size-dimorphism with females tending to be larger than males (Shine 1994). Their diet is primarily earthworms, although they do consume leeches (Carpenter 1952). Butler’s Gartersnakes brumate (akin to hibernate) communally during the winter months, and mate in groups after emerging from brumation in spring (Rossman et al. 1996). They give birth to live young during the summer or early fall and are not known to provide any form of parental care (Shine 1988; Rossman et al. 1996). Research on the female movement patterns has found that their home range is ~0.9 hectares (9000 m^2^) with daily short-distance travel and a maximum travel distance during their active season of 183 ± 32 m (Shonfield et al. 2019). Less is known about the movement of male Butler’s Gartersnakes, but captures at artificial shelters indicate an average maximum movement distance of 65.3 m (Shonfield et al. 2019).

### Study population, study area, and sampling

Data were obtained from a capture-mark-recapture study initiated to assess a population of Butler’s Gartersnakes under threat of a road construction project ~2 km east of the Detroit River near Windsor, Ontario, Canada. Snakes were monitored from 2009 to 2020. Construction of the road ran from 2011 to 2015 during which a temporary snake fence barrier was erected to prevent snakes from entering the construction zone. During the construction period, snakes captured in the construction zone were relocated to areas ~50 m beyond this zone. Following construction, a permanent barrier was installed around the road and survey efforts outside the road footprint continued until 2020. In total, 3801 individual Butler’s Gartersnakes were sampled during the 12-year study. We excluded 338 individuals due to missing information that was necessary for our analyses (i.e., sex, weight, location of capture, time of capture, and length). Thus, the total number of snakes considered in this analysis was 3463 individuals (53.3% Female; 46.7% Male). The study area was approximately 250 ha in size and contained three major sampling zones (Supplementary Figure S1) and two minor zones (the road and an ecopassage). There were no transitions between major zones in snakes that were recaptured. Coverboards (½ or ⅝ inch plywood) measuring 0.84 × 1.22 m were deployed to attract and facilitate the capture of Butler’s Gartersnakes. The coverboards were deployed from 1 April to 31 October in areas of suitable habitat. Some existing debris such as wood planks or tin sheets were also regularly inspected during coverboard surveys. All coverboards deployed in a given year were surveyed once per week and surveys were conducted during the 3 h before sunset and the 3 h following sunrise. Given the importance of relocating Butler’s Gartersnakes before major clearing and construction began, a higher density of coverboards was deployed in the construction zone in 2011 compared to all other construction years to support relocation efforts. The number of coverboards deployed in the construction zone from year to year was highly variable due to construction activities (2011:794, 2012:494, 2013:4, 2014:74). Conversely, coverboard densities outside the construction zone were kept relatively constant across years but still varied due to evolving study objectives and human disturbance of boards (2011–2019 average ± SD: 670 ± 118 coverboards; range: 441–778). Additionally, to increase the capture rate for the relocation effort, pitfall and funnel traps were also deployed at entrances to the construction zone from 2012 to 2014.

Captured snakes were weighed to the nearest 0.1 g (PESOLA spring scale 500 g), measured (total length and tail length), as well as sexed by probing (Laszlo 1975), and visually by comparing tail length to snout to vent length (King 1989). Both methods were used concurrently to ensure accuracy. The collected mass and length values were used to calculate body condition using the scaled mass index which provided values comparable to a residual regression method (Peig and Green 2009). Snakes that were under 20 g were given unique ventral scale clippings, while snakes over 20 g were implanted with an RFID PIT tag (Biomark MiniHPT10). Following capture, the snakes were transported to a nearby processing lab. Although most snakes were released one to two days following capture, animal care protocols required that some be released up to 10 days following their capture (e.g., if they were injured or gave birth in captivity).

### Social network analysis

Social networks were used to quantify grouping patterns. The networks were built in R v4.2.1 (R Core Team 2022) using the *igraph* package (Csárdi et al. 2023). In the networks, we quantified connections between snakes (i.e., edges) as the probability of social association based on the temporal and spatial proximity of snakes at the time of capture. We combined all capture data into a 12-year network with associations temporally and spatially thresholded. We used different thresholds to create variations of our network with edges quantified at a broad and precise scale level of association. To quantify the broad scale (Br) network, the edges were gradients of combined temporal and spatial proximity. Probabilities were calculated using a maximum temporal proximity of 14 days and a maximum spatial proximity of 50 m. In other words, if a snake was found within 50 m of another snake, and within 14 days, that was considered an association. Edges represented the probability of association with individuals found at the same place (i.e., same easting and westing) on the same day having an edge weight of 1 (representing a 100% chance of association). Individuals found further apart (spatially or temporally) had probability edge weights less than 1. Any individuals that were more than 14 days apart or more than 50 m apart were deemed to have a probability of association (and edge weight) of zero. Fifty meters was chosen based on previous research on Butler’s Gartersnake movement patterns (Shonfield et al. 2019) and the observed effects were tested for sensitivity to changes in this threshold (Supplementary Table S1). Fourteen days was based on the keen scent perception capabilities of snakes (Halpern and Martínez-Marcos 2003). More specifically, 14 days was chosen based on the only scent trail longevity study that we were aware of in which 70% of male African Brown House Snakes (*Boaedon fuliginosus*) could still follow a female scent trail that was 14 days old (Wilmes et al. 2012). However, due to a lack of research on the longevity of social-cue perception in gartersnakes, we also tested the robustness of the finding from the 14-day broad scale network (Br-14) by replicating the analyses with association thresholds of 10 days (Br-10) and 5 days (Br-5). For reporting purposes, we refer to this process as temporal thinning.

For the precise scale (Pr), all snakes were included in the network, but the parameters for what constituted an association changed to only those snakes with an edge weight of 1 (i.e., in the same place at the same time). In this case, there were far fewer associations as snakes were infrequently found at the same place on the same day. Instead, snakes were more commonly found by themselves (62% of observations). When a group of snakes were found at the same place at the same time, it was rarely the same set of individuals. In fact, 96% of dyads (i.e., two individuals found together) were unique pairs. Repeat dyads were rare primarily because repeat captures were rare, with most snakes (65%) being captured only once across the 12-year study period (*M*_captures_ = 1.73, SD ± 1.39). If snakes were captured more than once, weight and snout-vent-length were averaged for the analysis and year of capture was taken as the first year. We quantified sociability as an individual’s summed edge weight (summed probability of associations). Therefore, snakes with higher sociability spent more time near other snakes. To test if direct associations (the precise level) occurred more than would be expected by chance, we tested the number of observed dyads against two null models with modified data permutations that controlled for space use. Null models that control for space use have been used to demonstrate non-random social patterns in social network analysis (Spiegel et al. 2018; Tichon et al. 2020). For one null model, we held location and year of capture constant and allowed day of capture within the month to vary. In other words, in the random network, snakes chose the same location without coordinating time. For the second null model, we held time of capture constant and permuted the observed location of each snake within each capture zone. In this null model, snakes chose a different shelter in their zone on the same day. We then compared the number of observed dyads to the number of dyads generated under 10,000 iterations of the null models.

To further characterize snake grouping patterns, we tested for sex and age homophily. Homophily occurs when individuals are more likely to associate with similar individuals (Majolo and Huang 2017). Homophily is often quantified with a value that ranges from 1 to −1. A value of 1 means that individuals within the network only associate with similar individuals and a value of −1 means that individuals only associate with dissimilar individuals (Newman 2003). We used homophily as evidence for sex and/or age segregation in our networks as the two concepts are intrinsically related (Henry et al. 2011; Melamed et al. 2020). To test the significance of the homophily values, we compared the computed values to the values calculated from 10,000 networks in which the vertex characteristic of interest was permuted. In addition to homophily, we subdivided each network into communities of individuals that associated with each other. We used a Louvain partition for community detection (Blondel et al. 2008). To test the significance of the communities, we compared their robustness to random network communities using the *robin* R package (Policastro et al. 2021). Robin provides a Bayes factor (BF) which is a likelihood ratio of evidence for the alternative versus the null hypothesis. A Bayes factor greater than 1 indicates evidence favoring the alternative hypothesis, and a Bayes factor of 3 would indicate that the alternative hypothesis is 3 times more likely than the null (Schmalz et al. 2023). For this analysis, the alternative hypothesis was that observed community robustness was different from the robustness of communities generated from random graphs. The null hypothesis was that there was no difference in robustness.

To understand the importance of individuals within the communities, we calculated the vertex betweenness centrality (betweenness) of the members. We note that clustering individuals into communities changed the connectivity of the network by removing between community connections. Therefore, the betweenness analysis identified the characteristics of snakes that connected individuals within subgroups of the overall network (Farine and Whitehead 2015). More specifically, individuals with high betweenness scores were part of more “shortest paths” between other individuals in their communities (Freeman 1979; Brandes 2001).

### Statistical analysis

We tested for the effects of sex, age/weight, and body condition on sociability and community betweenness values. Collinearity between predictors was low (all variance inflation factors < 1.3). As age is a function of size in gartersnakes, we used weight as a proxy for age in all analyses (Shine and Charnov 1992; Feldman and Meiri 2012). Weight is a relatively good predictor of sexual maturity in snakes, and snakes continue to grow in adulthood; however, growth eventually plateaus (Shine and Charnov 1992). Body condition was calculated using the scaled mass index with a robust linear regression (Peig and Green 2009). Notably, the scaled mass index was designed to better reflect body condition compared to other common measures; however, it is still derived from length and mass and will fluctuate with changes to either (Peig and Green 2009). To assist in model convergence, body condition values were log_2_ transformed and weight values were scaled. Due to a large number of individuals without scores (isolated individuals for sociability and/or individuals who were part of no shortest paths for betweenness), distributions of scores were zero inflated. To deal with zero inflation, we used hurdle models to analyze sociability and betweenness scores (Feng 2021). As a result, analyses were divided into two models. One model was a binary logistic regression that looked for differences between individuals that had a score (all positive values collapsed to a value of 1) and individuals that did not have a score (a value of zero). This part of the analysis identified which factors contributed to overcoming the “hurdle” (i.e., to what extent did sex, age, or body condition make a snake more likely to have a positive/non-zero score). The second model looked for differences between individuals with positive scores and all zero scores dropped. This part of the analysis was concerned with the important factors influencing differences in the magnitude of scores once the “hurdle” of having a score has been crossed. The positive value models used a gamma distribution with a log link. For logistic regressions, we reported the odds ratios (OR) and their respective confidence intervals (CI). For the gamma distribution models, we reported the exponentiated slopes and their confidence intervals. We used the R package lme4 (Bates et al. 2015) to fit generalized linear mixed effect models using capture location and capture year as random effects.

To test models, we progressively added fixed effects and compared the Akaike information criterion (AIC) values of each model. AIC is a statistical tool for model comparisons that balances model fit and simplicity by penalizing goodness of fit values for added terms (Cavanaugh and Neath 2019). We considered models within two AIC values to be similar in fit (Cavanaugh and Neath 2019). As body condition is partially dependent on weight (Peig and Green 2009), we first compared a model with body condition to a model with weight and chose the variable that produced the better fit model. After we had determined if weight or body condition was a better fit for each dependent variable, we progressively added sex, then weight or body condition, then a sex by weight or sex by body condition interaction. In all models, we controlled for sampling across years by adding the covariate “number of years sampled” in any network. Although most models converged with the covariate and both capture location and capture year as random effects, some models required simplification for convergence. In these situations, we prioritized keeping the covariate over the random effects. For one model, it was necessary to remove a random effect and for another, the covariate for convergence (Supplementary Tables S2 and S3). We tested the obtained coefficients from the best fit model against the coefficients obtained from 10,000 node permutation models with swaps confined to capture zones within capture year. In all cases, we reported the more conservative *P*-values. For the reported models, we tested the type I error rate. To do so, we replicated the reported analyses 10,000 times with randomly generated independent variables (i.e., sex, weight, body condition) to identify the propensity with which the data generated type I errors (Farine and Carter 2021). For our models, type I error rates were low (range: 0.047–0.056; Supplementary Tables S2 and S3). For any model with a simulated type I error rate above 0.05, we applied a *P*-value correction (*P*_corrected_ = *P*_obtained_ × [α_simulated_/ α_expected_]). Uncorrected *P*-values can be found in Supplementary Tables S2 and S3.

Beyond these hurdle models, we performed two additional analyses to further parse the relationship between demographic factors and social interactions. First, we tested for a relationship between zone usage and observed association patterns. To do so, we used a multinomial logistic regression with the major zones of capture as the dependent variable and sex and weight as independent variables. Additionally, as the broad scale analyses included temporally ordered associations, we tested for age and sex differences in the order of arrival in shared space. In other words, we looked for “leaders” and “followers” when space was shared. To avoid the possibility of counting the offspring of mother snakes, we only used the pre-birth months of March to June (Rossman et al. 1996). We then designated snakes as leaders and followers based on the order of shared space occupancy and performed a binomial logistic regression with dyad as a random effect using the *brms* package (i.e., a multi membership model; Rushmore et al. 2013; Boyland et al. 2016; Bürkner 2017). For these models, we report credibility intervals (CrI) and *P*-values derived from the *bayestestR* package (Makowski et al. 2019).

## RESULTS

### Sociability

Direct interactions between snakes occurred more often in our observed data than in either permuted null models (location held constant and time varied; *P* < 0.001; time held constant and location within zone varied; *P* < 0.001). This suggests that association patterns were non-random.

**Figure 1:**
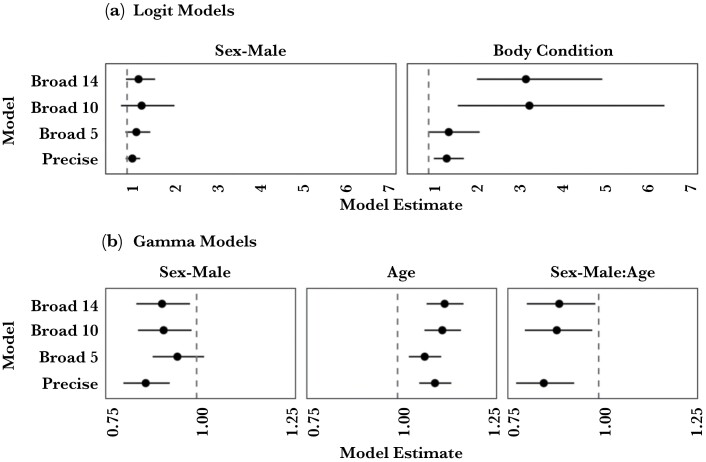
Model estimates and confidence intervals for the fixed effects of sex, age, and body condition in the Logit (A) and Gamma model (B) components of the sociability hurdle models. The y-axis shows the scale by which associations between snakes are constrained in the model (see text for details). On the x-axis, estimates for Sex-Male, Age, or Body Condition above 1 indicate that males, older snakes, or snakes with better body condition are more likely to have a sociability score (A) or have larger sociability scores (B) than females, younger snakes, or snakes with worse body condition respectively. Values below 1 indicate the opposite. Models were selected by AIC, so not all models contain all effects.

We used hurdle models (see statistical analyses) to further explore patterns of sociability. For the sociability logit models, body condition was a better fit than age for the presence or absence of a social connection (Figure 1A). At both the broad scale (Br14) and precise scale (Pr) network levels, there was a significant main effect of body condition (BC) such that individuals with higher body condition scores were more likely to be part of the network (i.e., have a connection with at least one other snake) (main effect BC; Br14; OR = 3.28, CI[2.13, 5.07], *P* = < 0.001; Pr; OR = 1.42, CI[1.12, 1.83], *P* = 0.004). At both network levels, males were more likely to be connected to the network but this effect was non-significant (no main effect of Sex; Br14; OR = 1.27, CI[0.97, 1.66], *P* = 0.077; Pr; OR = 1.12, CI[0.97, 1.31], *P* = 0.135). Temporal thinning of the broad scale network to 10 and 5 days had little effect on these trends (Figure 1A). These were the only models in which body condition was a better predictor than age. A multinomial logistic regression found no relationship between body condition and capture location (χ^2^_2_ = 2.86, *P* = 0.239).

**Figure 2:**
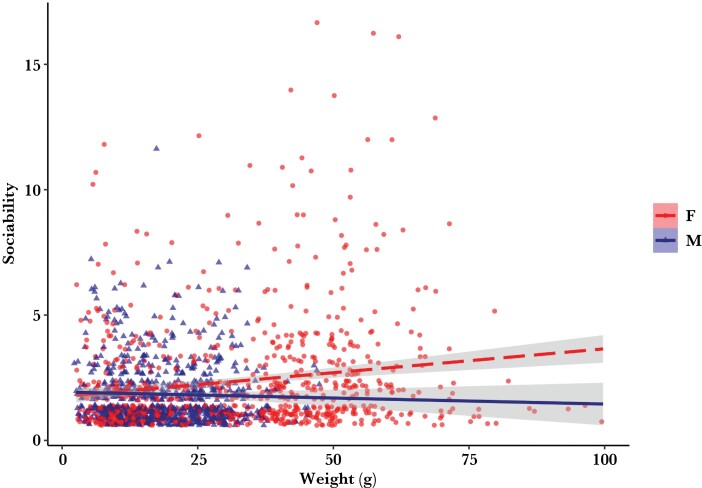
Raw data with regression lines showing Butler’s Gartersnake sociability scores as a function of sex and weight at the precise scale network level. Social score (sociability) is the cumulative number of dyadic interactions at the precise scale level of analysis. Female snakes are shown as filled circles and the dashed line. Male snakes are shown as triangles and the solid line. Shading represents 95% confidence intervals. The regression line for male snakes is extended for readability and caution should be used when interpreting values greater than 50 g for male snakes.

For the magnitude of sociability scores, at both the broad and precise scale network levels, the best models had an interaction effect between sex and age. The interaction was such that the relationship between age and sociability in males was reduced compared to the same relationship in female snakes (Br14; β = 0.89, CI[0.80, 0.99], *t* = −2.13, *P* = 0.004; Pr; β = 0.85, CI[0.77, 0.93], *t* = −3.41, *P* < 0.001). More specifically, as female snakes increased in age, they also tended to increase in sociability, while the opposite pattern occurred in male snakes (Figure 2).

At both scales, the best models also contained significant main effects of sex (Br 14; β = 0.90, CI[0.83, 0.98], *t* = −2.4, *P* = 0.005; Pr; β = 0.86, CI[0.80, 0.93], *t* = −3.99, *P* < 0.001) and age (Br 14; β = 1.13, CI[1.08, 1.18], *t* = 5.31, *P* < 0.001; Pr; β = 1.1, CI[1.06, 1.15], *t* = 4.8, *P* = 0.007). These effects should be interpreted with caution because of an interaction. Temporal thinning had no effect on the trends or significance when interactions were constrained to 10 days, but changed the significant effects at 5 days (Figure 1B; Br 10; interaction effect; β = 0.88, CI[0.80, 0.98], *t* = −2.29, *P* = 0.002; main effect of sex; β = 0.91, CI[0.84, 0.99], *t* = −2.30, *P* = 0.006; main effect of age; Br 10; β = 1.12, CI[1.07, 1.17], *t* = 5.09, *P* < 0.001; Br5; interaction effect was not part of the best model; no main effect of sex; β = 0.95, CI[0.88, 1.02], *t* = −1.42, *P* = 0.158; main effect of age; β = 1.07, CI[1.03, 1.12], *t* = 3.41, *P* < 0.001). Number of years sampled was significant in all models at all levels (Supplementary Table S2).

### Homophily

We found significant sex homophily at both the broad scale (*r* = 0.12, *P* < 0.001) and precise scale (*r* = 0.20, *P* < 0.001) network levels. At both levels, same-sex associations were more likely between females than between males (Table 1). There was also significant age homophily in associations. Similar to the sex homophily, the effect of age was stronger in the precise scale network (*r* = 0.51, *P* < 0.001) than in the broad scale network (*r* = 0.26, *P* < 0.001). Temporal thinning of the broad scale network had little impact on the sex (Br10; *r* = 0.12, *P* < 0.001; Br5; *r *= 0.12, *P* < 0.001) or age homophily values (Br10; *r* = 0.28, *P* < 0.001; Br5; *r* = 0.27, *P* < 0.001; Supplementary Table S4).

**Table 1:** Mixing matrix by sex showing the proportion of same-sex and opposite-sex connections in the network

	Female	Male
Broad scale network (14):		
Female	0.33	0.22
Male	0.22	0.23
Precise scale network:		
Female	0.40	0.19
Male	0.19	0.22

A multinomial logistic regression with capture zone as the dependant variable found a significant effect of age on capture zone occupancy (χ^2^_2_ = 104.09, *P* < 0.001) with older individuals slightly more likely to be found in two of the three capture zones (compared to Zone 1; Zone 2 OR = 1.03, CI[1.02, 1.04]; Zone 3 OR = 1.026, CI[1.02, 1.03]). Although males were also more likely to be found in zone 2 and 3 (compared to Zone 1; Zone 2 OR = 1.36, CI[0.99, 1.86]; Zone 3 OR = 1.04, CI[0.89, 1.22]), the overall effect of sex was not significant (χ^2^_2_ = 3.07, *P* = 0.157). This suggests that age homophily may be driven by differences in zone use.

### Communities and centrality

Excluding isolated snakes, the average community size for the precise network was 3.56 with high variance (SD = 4.86). Community sizes ranged from 2 to 46. Increasing the scale inevitably increased the size of the communities. At the broad scale (14), the average community consisted of 20.4 snakes (SD = 41.24) with the largest community consisting of 271 snakes. At both scales, we found that communities were much more robust than would be expected in a random network (Br14; BF = 314.36; Pr; BF = 276.5). Temporal thinning of the broad scale network had little effect on community robustness (Br10; BF = 322.68; Br5; BF = 309.1). Based on the Bayes factor interpretations provided by Jeffreys (1961), there is decisive evidence that Butler’s Gartersnake communities are non-random.

**Figure 3:**
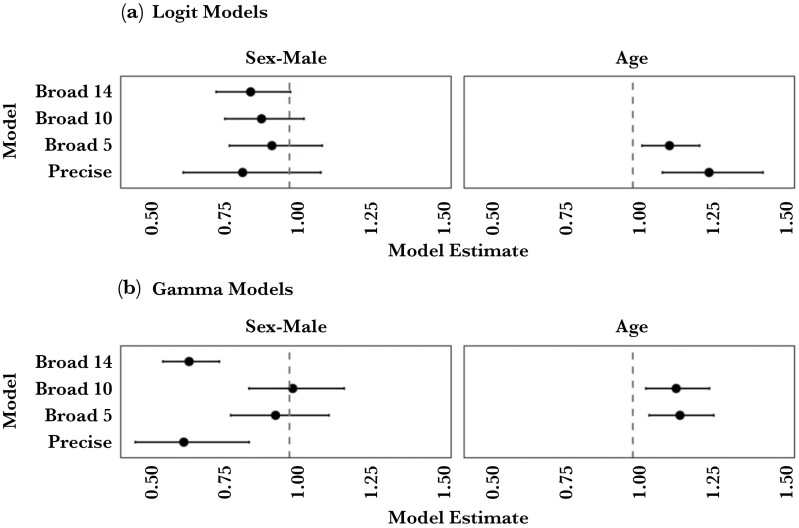
Model estimates and confidence intervals for the fixed effects of sex and age in the Logit (A) and Gamma model (B) components of the betweenness hurdle models. The y-axis shows the scale by which associations between snakes are constrained in the model (see text for details). On the x-axis, estimates for Sex-Male and Age above 1 indicate that males or older snakes are more likely to have a betweenness score (A) or have larger betweenness scores (B) than females or younger snakes respectively. Values below 1 indicate the opposite. Models were selected by AIC, so not all models contain all effects.

We divided analysis of community betweenness (i.e., centrality) scores into logit models looking at the presence or absence of a betweenness score, and models only looking at the magnitude of positive scores. For the logit models, age was a better predictor than sex for community betweenness (Figure 3A). To elaborate, at the broad scale level of analysis, the best model contained a marginal effect of sex with males being less likely to have a betweenness score in their communities (Br14; OR = 0.87, CI[0.75, 1.00], *P* = 0.056). This effect was not found at any other scale of analysis (no main effect of sex; Pr; OR = 0.84, CI[0.64, 1.11], *P* = 0.219; Br5; OR = 0.94, CI[0.80, 1.11], *P* = 0.172; Br10; OR = 0.91, CI[0.78, 1.05], *P* = 0.187). At both the precise level and the most constrained broad scale level (Br5), the best model had a significant effect of age with older snakes more likely to have a betweenness score in their community (Pr; OR = 1.27, CI[1.1, 1.44], *P* < 0.001; Br5; OR = 1.12, CI[1.03, 1.23], *P* = 0.008). Although age was not a significant factor in broader scale networks, removal of the “number of years sampled” control variable from these analyses found that it was obfuscating the effect of age identified in the other models (Br14; OR = 1.20, CI[1.12, 1.30], *P* < 0.001; Br10; OR = 1.22, CI[1.13, 1.33], *P* < 0.001). To summarize, older snakes are more likely to have betweenness scores in their communities, likely because of increased connectivity both within and between years.

For the magnitude of betweenness score models, either sex or age was the important predictor depending on the scale of the network (Figure 3B). The best model for both the broad scale network and the precise scale network had only a significant effect of sex with males tending to have lower betweenness values in their communities (Br14; β = 0.66, CI[0.57, 0.76], *t* = −5.64, *P* < 0.001; Pr; β = 0.64, CI[0.48, 0.86], *t* = −2.93, *P* = 0.008). Thinning the broad scale network removed the significant effect of sex (Br10; β = 1.01, CI[0.86, 1.19], *t* = 0.14, *P* = 0.37; Br5; β = 0.95, CI[0.80, 1.13], *t* = −0.54, *P* = 0.434), and added a significant effect of age with older snakes having larger betweenness scores (Br10; β = 1.15, CI[1.04, 1.26], *t* = 2.86, *P* = 0.002; Br5; β = 1.16, CI[1.05, 1.28], *t* = 3.08, *P* = 0.041). Removal of the number of years sampled variable revealed the significant effect of age at the broadest scale (Br14; β = 1.21, CI[1.12, 1.32], *t* = 4.55, *P* < 0.001) but not at the precise scale (Pr; β = 1.04, CI[0.86, 1.25], *t* = 0.40, *P* = 0.692). In other words, for snakes sharing the same space at the same time, females tended to have larger betweenness scores than males. At broader levels, older snakes tended to have larger betweenness scores than younger snakes.

To further understand the influence of sex and age on the nature of associations across time, we looked for patterns in the order of arrival when snakes were found at similar locations at different times from March until June (i.e., pre-birth months). There was a significant effect of age on order of arrival in shared space, with older snakes more likely to occupy a location first (Br14; OR = 1.13, CrI[1.10, 1.16], *P* < 0.001; Br10; OR = 1.09, CrI[1.05, 1.12], *P* < 0.001). Across broad scale networks, there was no effect of sex in order of arrival in shared space (Br14; OR = 1.00, CrI[0.95, 1.05], *P* = 0.987; Br10; OR = 0.99, CrI[0.93, 1.05], *P* = 0.912; Br5; OR = 0.96, CrI[0.83, 1.1], *P* = 0.823). The relationship between age and order of occupancy was non-significant when associations were constrained to 5 days (Br5; OR = 1.06, CrI[0.99, 1.14], *P* = 0.276). Significance and age trends were the same when all months were considered. To summarize, when snakes used the same space but at different times, older snakes tended to arrive first and younger snakes followed.

## DISCUSSION

We inferred social patterns based on the physical and temporal proximity of wild Butler’s Gartersnakes captured during a 12-year capture-mark-recapture study. We used social network analysis and looked at sociability and homophily in associations. We defined our social networks at multiple levels, with associations occurring at the same place on the same day (the precise scale level of analysis), and with associations defined as a snake’s probability of interaction with conspecifics within 50 m and 14 days (the broad scale level of analysis). We also tested for the effects found in the broad scale 14-day network with the probability of an interaction constrained to 10 and 5 days. In addition, we subdivided the networks into communities and looked at betweenness centrality within these communities. We hypothesized sex- and age-assorted social structures in Butler’s Gartersnakes and that network membership would offer benefits reflected in improved body condition. Based on recent laboratory research showing age and sex differences in sociability in gartersnakes (Skinner et al. 2022), we hypothesized that we would see the same differences in wild snakes and that differences in sociability could be driving network structures. Generally, we found support for our hypotheses. We found that snakes were more likely to associate with each other than expected by chance and our homophily analysis found that they tended to associate with individuals of the same age and that females tended to associate with each other. In other words, the general social structure consisted of age- and sex-assorted communities. Although it is difficult to completely rule out the effect of fine scale habitat differences on social interactions, we found little evidence that the observed communities were due to differences in habitat use in adults. However, we did find that younger snakes were more likely to be captured at one of the sampling sites.

When modeling the relationship between demographic factors and network measures (i.e., sociability and community centrality), we found that sex and age were important predictors of association patterns while better body condition predicted network membership. To elaborate, for sociability at all scales of analysis, older females were more social and older males were less social. When examining betweenness centrality in gartersnake communities, we found that both sex and age were important predictors of centrality, but the results were more sensitive to the temporal limits placed on associations. To further parse the centrality findings, age was important for having a betweenness score when associations were highly constrained (precise and broad scale 5-day) but not at broader scales of analysis. When considering the magnitude of centrality scores, both sex and age were important with either females or adults tending to be highly central at different levels of analysis. Overall, the volatility of the betweenness analyses suggests that there may be multiple processes affecting how snakes associate and share space across time.

### Social patterns: body condition

Despite finding that males and females differed in sociability, both sexes tended to be healthier (i.e., have better body condition) when they were part of the overall network relative to snakes that were never found with or near other snakes. This occurred irrespective of the level of network analysis (broad or precise) and suggests that both sexes may derive benefits to being near other snakes. Despite the common perception that snakes are non-social (Brattstrom 1974; but see Doody et al. 2013), it is well documented that snakes aggregate (Dundee and Miller 1968; Heller and Halpern 1982; Skinner and Miller 2020). Numerous benefits have been hypothesized for grouping including protection from predation (Graves and Duvall 1995), thermoregulation (Aubret and Shine 2009), moisture conservation (Noble and Clausen 1936), and information gained through the presence of conspecifics (Graves and Duvall 1988). Along with benefits, direct and indirect association between conspecifics can also have drawbacks such as resource competition (Stamps 1977; Yeager and Burghardt 1991), and the spread of disease and/or parasites (Drewe and Perkins 2014; Sih et al. 2018). Increased body condition in associating snakes suggests that some individuals may be better at exploiting the benefits and limiting the drawbacks of shared space use than others. Although some snakes may become healthier through social connectivity, these results are correlational; as such, the reverse could also be true with healthier individuals being more socially tolerant due to a competitive advantage.

While healthy individuals may balance competition against benefits, individuals with reduced body condition may struggle to compete for resources and therefore may avoid other snakes. Avoidance of resource competitors is a common strategy in nature (Weckerly 1998; Castelo et al. 2003; Szymkowiak et al. 2016), and research on reptile behavior suggests that intra-species competition can moderate social attraction (Plath et al. 2006; Riley et al. 2017). Alternatively, individuals with reduced body condition may be more likely to have parasites or disease and may thus avoid or be avoided by conspecifics (Stockmaier et al. 2021). Finally, although snakes did not differ in body condition across our sampling zones, it is difficult to rule out the possibility that some resource-scarce areas have both fewer and less healthy snakes.

Irrespective of the cause, the relationship between body condition and network membership is intriguing as social connectivity has been found to be important for fitness in group living animals (Ellis et al. 2019). In snakes, improved body condition can benefit male mating success (Shine et al. 2000) and increase female attractiveness (Shine et al. 2003). This suggests that group membership could also indirectly improve reproductive outcomes by supporting body condition. In summary, being part of the network may support the health of group members through a variety of benefits. Individuals may also leave the group if they cannot compete. It is important to note that general network membership was the only situation in which body condition was the best predictor of social behavior. In all other models, different factors influenced social structure to a greater extent.

### Social patterns: sex and age

Within the network, we found sex and age differences in the number and/or strength of social connections (i.e., sociability) irrespective of the scale of analysis. The prevailing pattern of interaction was that older females tended to be more social while older males tended to be less social. Recent laboratory research on ontogenetic changes in gartersnake sociability found the same pattern; as female snakes increased in age, they tended to be more attracted to a social stimulus derived from conspecific skin lipids. In contrast, as male snakes aged, they tended to be less attracted to conspecific skin lipids (Skinner et al. 2022). This suggests that these similar patterns in wild Butler’s Gartersnakes are driven by innate changes in social attraction or learned patterns from social interactions. Either way, these combined results suggest that male snakes are more sensitive to competition as they age. Due to the importance of size in male mating success (Shine et al. 2000), sexually mature male snakes may attempt to reduce direct competition through social avoidance. To some extent, male avoidance of females is likely supported by mostly confining opposite sex associations to seasonal mating at den sites (Rossman et al. 1996). Interestingly, female affiliation and male avoidance has also been found in both laboratory and radiotelemetry observations of Cottonmouths (Roth and Lutterschmidt 2011), and in a radio telemetry study of Eastern Brown Snakes (Whitaker and Shine 2003), which implies that these patterns may be widespread across snake species.

### Homophily

Homophily often occurs in social networks (McPherson et al. 2001), and sex- and age-homophily occurs in humans (McPherson et al. 2001) and non-human animals (Bottlenose Dolphins, *Tursiops* spp.; Lusseau and Newman 2004; Barbary Macaque, *Macaca sylvanus*; Sosa 2016). We found significant levels of sex- and age-homophily in our networks. Direct comparisons of data from other species to our results are difficult, as patterns of homophily remain relatively untested in snakes. However, indirect evidence suggests that it occurs in some species. In Timber Rattlesnakes, juveniles and related pregnant females hibernate in close proximity, suggesting both age- and sex-homophily (Clark et al. 2012). In a field experiment looking at patterns of aggregation under covered objects, Gregory (2004) found that snakes of similar size were often found hiding together, suggesting age homophily.

Inspection of the sex homophily mixing matrix (Table 1) suggests that the sex homophily found in the networks may be driven by social attraction in females (discussed above). We found little evidence that sex homophily was linked to differences in space use. However, we hesitate to rule out this possibility, due to our coarse measure of habitat use. In contrast to sex homophily, our analysis of capture zone biases found that young snakes were more likely to be captured at one of our sampling areas. This suggests that age segregation may be partially due to habitat partitioning. As primarily earthworm specialists, partitioning is unlikely due to differences in prey. Instead, partitioning may be the result of gartersnake females choosing particular ideal birthing locations (Graves and Duvall 1995) possibly due to differences in soil moisture (Shonfield et al. 2019). Preferred birthing locations in combination with delayed or relatively short-distance neonate dispersal (e.g., Broad-headed Snakes; *Hoplocephalus bungaroides*; Webb and Shine 1997; Grey Ratsnakes; *Patherophis spiloides*; Blouin-Demers and Weatherhead 2021) could result in age homophily due to site occupancy patterns.

Ontogenetic changes in habitat usage have been recorded in other squamates (Bronze Anole; *Anolis aeneus*; Stamps 1983; *Trioceros jacksonii xantholophus*; Jackson’s Chameleon; Van Kleeck et al. 2018) including Oregon Gartersnakes (*Thamnophis atratus hydrophilus*; Lind and Welsh 1994). It is not possible with our data to dissociate to what extent affiliative behavior contributes to juvenile habitat choices in Butler’s Gartersnakes. However, considering juvenile gartersnakes are drawn to conspecific odor and readily aggregate (Heller and Halpern 1982; Graves and Halpern 1988; Lyman-Henley and Burghardt 1994; Skinner and Miller 2020), social attraction likely contributes in some way. Irrespective of the causes of the homophily that we identified, a likely consequence is segregation into communities (Lusseau and Newman 2004).

### Communities and community centrality

Our findings of significant homophily combined with distinct communities suggests that Butler’s Gartersnakes are sorting/segregating into sex- and age-based communities. Future research should determine if these are matri-focal/matrilineal communities. The link between homophily and segregation is well established and can arise in networks when there is even a weak tendency to avoid dissimilar individuals (Henry et al. 2011). In this way, demographic patterns of social attraction and avoidance result in homophily which in turn generates communities. Although sex and age-dependent shifts in sociability appear to be the likely proximate cause of the emergent communities, the ultimate cause is more likely linked to competition and/or differing resource needs between population subsets similar to what has been suggested in ungulates (Ruckstuhl and Neuhaus 2000), cetaceans (Wearmouth and Sims 2008), and other reptiles (Shine and Wall 2005). For example, females may be driven to associate by a mutual need to maintain a more precise body temperature than males (Shine and Wall 2005) which could drive females to particular locations and/or increase the thermoregulatory value of social contact. Either way, within gartersnake communities, individuals differ in their connectivity with age and sex playing an important role in determining the likelihood of a central position. Within communities, we found that female Butler’s Gartersnakes had higher betweenness centrality scores than males. Female centrality is a social-structure characteristic commonly found in animals in which females (often related) maintain strong bonds and males disperse (Wearmouth and Sims 2008; Harcourt 2010), such as cetaceans (Rendell et al. 2019) and some primates (Sosa 2016). Research on other snake species have suggested similar patterns with male-biased dispersal (Eastern Small-eyed Snake, *Rhinoplocephalus nigrescens*; Keogh et al. 2006; Slaty-grey Snake, *Stegonotus cucullatu*s; Dubey et al. 2008) and affiliative behavior among related females (Timber Rattlesnakes; Clark et al. 2012; Cottonmouths; Hoss et al. 2015). Differences in sociability may also contribute to differences in centrality similar to patterns seen in some primates in which social individuals also tend to be central (Cheney et al. 2016; Beisner et al. 2021). Research on gartersnake social behavior has shown consistent individual differences in sociability within a group (Skinner and Miller 2022), and higher sociability scores in older females (Skinner et al. 2022), both of which could directly support differences in betweenness centrality.

Along with sex, age was also important for community centrality with adults more likely to have a centrality score at more exact levels (precise and broad constrained to 5 days) and more likely to have larger centrality scores at some broad levels (5 days and 10 days). Increased social connectedness in adults can occur naturally in fission–fusion societies where individuals encounter more conspecifics over time (e.g., Giraffe, *Giraffa camelopardalis*; Lavista Ferres et al. 2021; African Elephant, *Loxodonta africana*; Wittemyer et al. 2005); the longer an individual survives, the more time it has to interact with others. In our models, we included increased sampling of adults across years as a control variable, and still found that older snakes tended to be more central at some levels of analysis. Increased centrality can also be the result of non-social factors such as movement patterns (Spiegel et al. 2016, 2017). In many animals, home range size increases with body size (Hendriks 2007) and this relationship also occurs in snakes (Todd and Nowakowski 2020). Therefore, adults may travel further than younger snakes which would bring them near more conspecifics. Increased connectivity could also be the result of ontogenetic changes in social behavior driven by sexual maturity. For example, gestating females will increase their social connections at birthing rookeries (Reichenbach 1983), and adult male snakes may try to remain near healthy breeding populations.

Along with these other possibilities, our data suggest that adult snakes may have increased centrality through sharing space with younger snakes. Our analysis found that young snakes tended to follow older snakes. This suggests that young snakes may take advantage of chemosensory social information from adults when choosing sheltering locations. This tendency to follow conspecific chemosensory cues has been reported in multiple species of snake (Ford 1986) including gartersnakes, which will use scent trails to find both mates (Ford 1982) and sheltering locations (Heller and Halpern 1982). If young snakes follow adults, then these adults may be social information bridges between individuals (i.e., have high centrality).

An unfortunate consequence of being a social information bridge is that these central individuals have a higher risk of becoming vectors for disease transmission (MacIntosh et al. 2012). This suggests that for infections such as snake fungal disease, adult females may be an indicator of more extensive transmission in gartersnake communities and an important point for potential intervention.

## CONCLUSIONS AND IMPLICATIONS

The benefits that Butler’s Gartersnakes may receive from group membership include thermoregulation, moisture retention, social information, and possibly reduced chances of predation (Graves and Duvall 1988, 1995; Aubret and Shine 2009). Our results add to literature suggesting that even with relatively few behaviorally simple grouping benefits, the patterns of sociality that emerge can be similar to those observed in animals often considered highly social (Halliwell et al. 2017). More specifically, our results suggest that sex-linked patterns in sociability that balance simple grouping benefits with competitor avoidance can result in basal social structures similar to those observed in other vertebrates. It is notable that Butler’s Gartersnakes give birth to live young, as a comparative phylogenetic analysis of squamate reptiles found that live birth was a significant predictor of social grouping (Halliwell et al. 2017). However, despite Butler’s Gartersnake social groups sharing structural features with other taxa, their groups appear to be less stable than those found in many group-living taxa, including other viviparous squamates (e.g., Skinks; *Scincidae*; Halliwell et al. 2017).

Although we report age and sex assorted social structure, we note that the nature of associations may change in ways that are undetectable by our analysis. Due to the cryptic nature of snakes, we have assumed that our network measures capture aspects of sociality without direct observation of their social interactions. Despite this assumption, our results align heavily with other findings from research on snake sociability (Roth and Lutterschmidt 2011; Skinner and Miller 2020; Skinner et al. 2022). It is important to recognize that interactions at broad and precise scales of analysis certainly differ. For example, when individuals share the same space at the same time (precise scale) interactions can involve direct aggregation benefits, whereas at broader scales, individuals could interact with chemosensory social information while actively avoiding direct contact. Our ability to understand interactions is also limited by the use of weight as a proxy for age, as weight eventually plateaus in snakes. Due to this limitation, different methodologies will be necessary to assess some age-dependent changes in social behavior. For example, whether or not an individual’s social network shrinks with senescence as seen in some social animals (Albery et al. 2022; Siracusa et al. 2022). Finally, due to the motivation for the original project, it was necessary to move snakes out of the construction area. Although we cannot completely rule out the possibility that this impacted their social interactions, the fact that we did not move snakes far and that they tended to show zone fidelity suggests that general social patterns likely remained stable.

Despite the challenges inherent in interpreting proximity based social behavior, it is important that efforts are made to quantify social patterns in cryptic animals as understanding social structures can improve conservation efforts (Slotow et al. 2000; Walker et al. 2009; Shier and Swaisgood 2011). Here, we demonstrated that data collected through capture-mark-recapture projects can be used to understand population-level social patterns in a species with cryptic social behavior. Importantly, many organizations have already collected capture-mark-recapture data that might be leveraged to the same ends. Hopefully, continued research on social interaction patterns can expand our understanding of snake social behavior and provide conservation managers with the necessary information to develop strategies that accommodate the needs of particular species and to different demographics within the species.

## Supplementary Material

arad095_suppl_Supplementary_Figures_S1_Tables_S1-S4

## Data Availability

Analyses reported in this article can be reproduced using the data provided by Skinner et al (2023).
