# Supplementary material for: Social networks reveal sex- and age-patterned social structure in Butler’s gartersnakes (Thamnophis butleri)
Source: Behav Ecol. 2023 Nov 14;35(1):arad095. doi: 10.1093/beheco/arad095 (PMC10773305; doi:10.1093/beheco/arad095)
Supplement: arad095_suppl_Supplementary_Figures_S1_Tables_S1-S4 [file arad095_suppl_supplementary_figures_s1_tables_s1-s4.docx]

**Article: Social Networks Reveal Sex- and Age-Patterned Social Structure in Butler’s Gartersnakes (*Thamnophis butleri*)**

**Authors: Morgan Skinner^1*^, Megan Hazell^2^, Joel Jameson^3^, Stephen C. Lougheed^2^**

**Author affiliations: 1. Wilfrid Laurier University, 2. Queen’s University, 3. WSP**

**Journal: Behavioral Ecology**

***corresponding author - e-mail address: skin4450@mylaurier.ca**


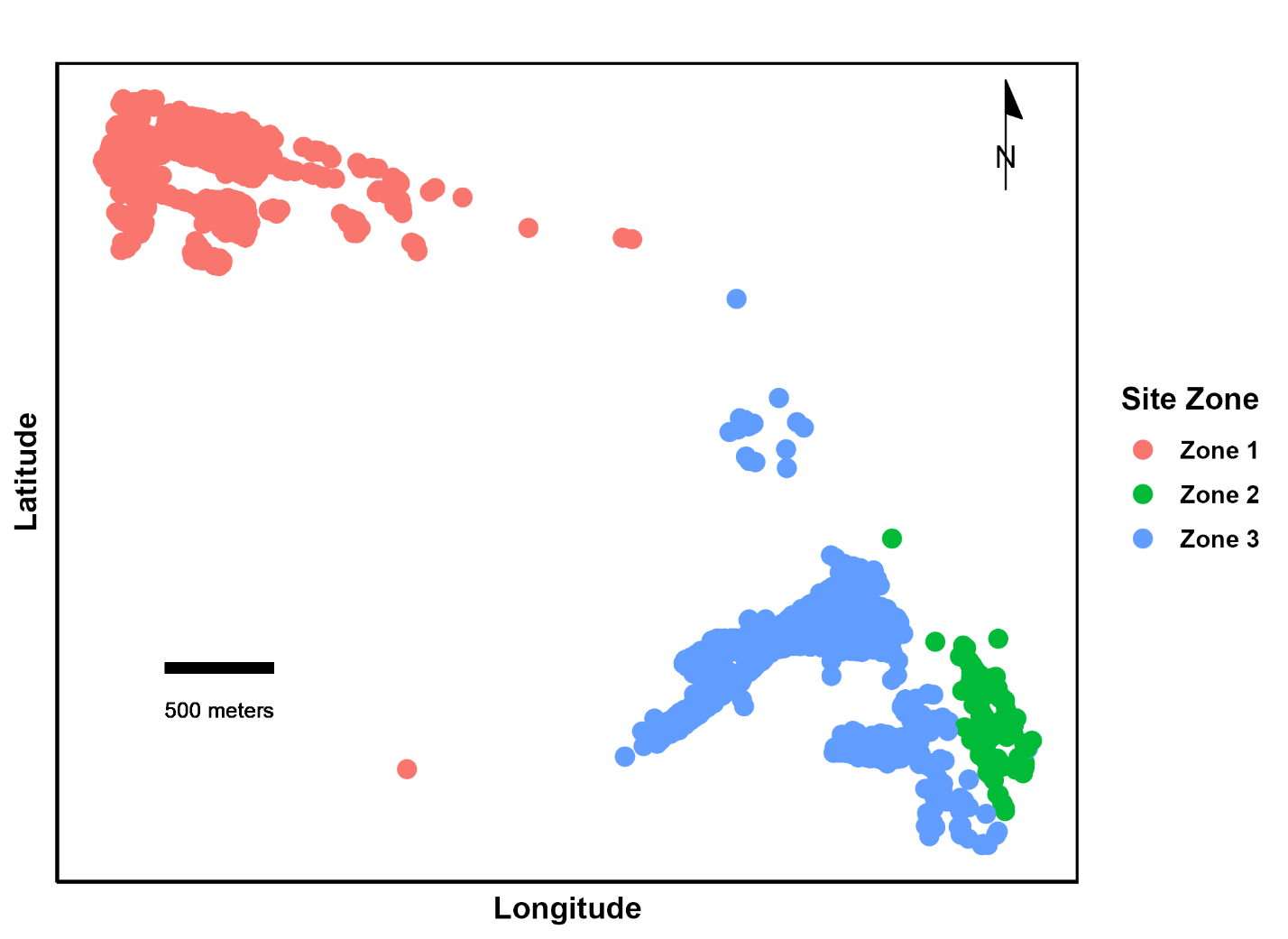


**Figure S1** Anonymized map of the snake capture locations at the study site. Dots represent snake captures colored by capture zone (see text for details). The background study site map has been removed but the relative positions of snake captures remain intact.

**Table S1** Stability of the significant fixed effects of the broadest scale model after changing the proximity criteria for an interaction to 25, 10, and 5 meters. Values are reported for both Sociability (I) and Betweenness (II) and are further subdivided into logistic regressions (A) and Gamma (B) models (see text for details). Broad (50m) is equivalent to Broad(14). The Number of Years Sampled (# Yrs) was held constant in permutated models.

| I. Sociability | | | | | | | | | | | | | | | | | | | |
| --- | --- | --- | --- | --- | --- | --- | --- | --- | --- | --- | --- | --- | --- | --- | --- | --- | --- | --- | --- |
| A. Logit models | | | | | | | | | | | | | | | | | | | |
|  | Broad (5m) | | | | | | | Broad (10m) | | | Broad (25m) | | | | Broad (50m) | | | | |
|  | OR | | CI | | | p | | OR | CI | p | OR | CI | | P | OR | CI | | p | |
| Fixed E |  | |  | | |  | |  |  |  |  |  | |  |  |  | |  | |
| # Yrs | 4.62 | | 3.3,6.6 | | | n/a | | 5.45 | 3.7,8.5 | n/a | 15.7 | 6,63.2 | | n/a | n/a | n/a | | n/a | |
| Sex | 1.22 | | 1,1.4 | | | .02 | | 1.22 | 1,1.5 | .03 | 1.02 | .8,1.3 | | .864 | 1.27 | .97,1.7 | | .08 | |
| BC | 1.76 | | 1.3,2.3 | | | <.001 | | 1.84 | 1.4,2.5 | <.001 | 1.61 | 1.1,2.4 | | .02 | 3.27 | 2.1,5.1 | | .001 | |
| Sx*BC | --- | | --- | | | --- | | --- | --- | --- | --- | --- | | --- | --- | --- | | --- | |
| Rand. E | Year & Cap. Zone | | | | | | | Year & Cap. Zone | | | Year & Cap. Zone | | | | Year & Cap. Zone | | | | |
|  | | | | | | | | | | | | | | | | | | | |
| B. Gamma models | | | | | | | | | | | | | | | | | | | |
|  | Broad (5m) | | | | | | | Broad (10m) | | | Broad (25m) | | | | Broad (50m) | | | | |
|  | e^β^ | | | t | | | p | e^β^ | t | p | e^β^ | t | P | | e^β^ | t | | p | |
| Fixed E. |  | | |  | | |  |  |  |  |  |  |  | |  |  | |  | |
| # Yrs | 1.53 | | | 9.97 | | | n/a | 1.59 | 10.29 | n/a | 1.81 | 12.86 | n/a | | 2.01 | 14.7 | | n/a | |
| Sex | .84 | | | -4.07 | | | <.001 | .86 | -3.28 | <.001 | .9 | -2.52 | .002 | | .9 | -2.40 | | .005 | |
| Age | 1.14 | | | 5.45 | | | <.001 | 1.15 | 5.62 | <.001 | 1.15 | 5.89 | <.001 | | 1.13 | 5.31 | | <.001 | |
| Sx*Age | .81 | | | -3.77 | | | <.001 | .82 | -3.38 | <.001 | .84 | -3.07 | <.001 | | .89 | -2.13 | | .004 | |
| Rand. E | Year & Cap. Zone | | | | | | | Year & Cap. Zone | | | Year & Cap. Zone | | | | Year & Cap. Zone | | | | |
| II. Betweenness | | | | | | | | | | | | | | | | | | |  |
| A. Logit models | | | | | | | | | | | | | | | | | | |  |
|  | | Broad (5m) | | | | | | Broad (10m) | | | Broad (25m) | | | | Broad (50m) | | | |  |
|  | | OR | | CI | | | p | OR | CI | p | OR | CI | | P | OR | CI | p | |  |
| Fixed E | |  | |  | | |  |  |  |  |  |  | |  |  |  |  | |  |
| # Yrs | | 3.72 | | 3,4.6 | | | n/a | 3.84 | 3.1,4.8 | n/a | 7.63 | 5.9,10.1 | | n/a | 5.94 | 4.6,7.9 | n/a | |  |
| Sex | | .81 | | .67,.96 | | | .02 | .81 | .68,.97 | .02 | .95 | .82, 1.1 | | .56 | .87 | .75,1 | .06 | |  |
| Age | | --- | | --- | | | --- | --- | --- | --- | --- | --- | | --- | --- | --- | --- | |  |
| Sx*Ag | | --- | | --- | | | --- | --- | --- | --- | --- | --- | | --- | --- | --- | --- | |  |
| Rand. E | | Year | | | | | | Year & Cap. Zone | | | Year | | | | Year | | | |  |
|  | | | | | | | | | | | | | | | | | | |  |
| B. Gamma models | | | | | | | | | | | | | | | | | | |  |
|  | | Broad (5m) | | | | | | Broad (10m) | | | Broad (25m) | | | | Broad (50m) | | | |  |
|  | | e^β^ | | | t | | p | e^β^ | t | p | e^β^ | t | | P | e^β^ | t | p | |  |
| Fixed E. | |  | | |  | |  |  |  |  |  |  | |  |  |  |  | |  |
| # Yrs | | 1.55 | | | 4.92 | | n/a | 1.73 | 6.57 | n/a | 2.12 | 8.97 | | n/a | 1.99 | 9.7 | n/a | |  |
| Sex | | .72 | | | -3.08 | | .007 | .85 | -1.7 | .107 | .8 | -2.49 | | .001 | .66 | -5.64 | <.001 | |  |
| Age | | --- | | | --- | | --- | --- | --- | --- | --- | --- | | --- | --- | --- | --- | |  |
| Sx*Age | | --- | | | --- | | --- | --- | --- | --- | --- | --- | | --- | --- | --- | --- | |  |
| Rand. E | | Year & Cap. Zone | | | | | | Year & Cap. Zone | | | Year & Cap. Zone | | | | Year & Cap. Zone | | | |  |

**Table S2** For the sociability models, the non-exponentiated slopes, uncorrected p-values, simulated type I error rates, and included random effects for the logit (A) and gamma (B) components of the hurdle models at the precise and broad scales. Dashes indicate that the fixed effect was not included in the model with the lowest AIC. N/a indicates that the value was not calculated. The value in brackets for the Broad networks is the threshold beyond which the probability of association becomes zero.

Sociability

| A. Logit models | | | | | | | | | | | | | |
| --- | --- | --- | --- | --- | --- | --- | --- | --- | --- | --- | --- | --- | --- |
|  | Precise | | | | Broad (5) | | | Broad (10) | | | Broad (14) | | |
|  | β | p_obt_ | | α_sim_ | β | p_obt_ | α_sim_ | β | p_obt_ | α_sim_ | β | p_obt_ | α_sim_ |
| Fixed E |  |  | |  |  |  |  |  |  |  |  |  |  |
| # Yrs | 1.03 | <0.001 | | n/a | 2.32 | <0.001 | n/a | 2.43 | .01 | n/a | n/a | n/a | n/a |
| Sex | .12 | .130 | | .052 | .2 | .104 | .05 | .29 | .11 | .051 | .24 | .08 | .049 |
| BC | .35 | .004 | | .051 | .39 | 0.055 | .051 | 1.21 | <.001 | .056 | 1.19 | <.001 | .055 |
| Sx*BC | --- | --- | | --- | --- | --- | --- | --- | --- | --- | --- | --- | --- |
| Rand. E | Year & Cap. Zone | | | | Year & Cap. Zone | | | Year & Cap. Zone | | | Year & Cap. Zone | | |
|  | | | | | | | | | | | | | |
| B. Gamma models | | | | | | | | | | | | | |
|  | Precise | | | | Broad (5) | | | Broad (10) | | | Broad (14) | | |
|  | β | | p_obt_ | α_sim_ | β | p_obt_ | α_sim_ | β | p_obt_ | α_sim_ | β | p_obt_ | α_sim_ |
| Fixed E. |  | |  |  |  |  |  |  |  |  |  |  |  |
| # Yrs | .27 | | <.001 | n/a | .60 | <.001 | n/a | .68 | <0.001 | n/a | .7 | <0.001 | n/a |
| Sex | -.15 | | <.001 | .052 | -.05 | .155 | .051 | -.09 | 0.006 | .048 | -.1 | 0.005 | .049 |
| Age | .1 | | .007 | .05 | .07 | <.001 | .052 | .12 | <0.001 | .047 | .12 | <0.001 | .049 |
| Sx*Age | -.16 | | <.001 | .05 | --- | --- | --- | -.12 | 0.002 | .054 | -.11 | 0.004 | .052 |
| Rand. E | Year & Cap. Zone | | | | Year & Cap. Zone | | | Year & Cap. Zone | | | Year & Cap. Zone | | |

**Table S3** For the betweenness models, the non-exponentiated slopes, uncorrected p-values, simulated type I error rates, and included random effects for the logit (A) and gamma (B) components of the hurdle models at the precise and broad scales. Dashes indicate that the fixed effect was not included in the model with the lowest AIC. N/a indicates that the value was not calculated. The value in brackets for the Broad networks is the threshold beyond which the probability of association becomes zero.

| Logit models | | | | | | | | | | | | |
| --- | --- | --- | --- | --- | --- | --- | --- | --- | --- | --- | --- | --- |
|  | Precise | | | Broad (5) | | | Broad (10) | | | Broad (14) | | |
|  | Β | p_obt_ | α_sim_ | β | p_obt_ | α_sim_ | β | p_obt_ | α_sim_ | β | p_obt_ | α_sim_ |
| Fixed E. |  |  |  |  |  |  |  |  |  |  |  |  |
| # Yrs | 1.17 | <.001 | n/a | 1.60 | <.001 | n/a | 1.74 | <.001 | n/a | 1.78 | <.001 | n/a |
| Sex | -.17 | .215 | .051 | -.06 | .172 | .05 | -.1 | .187 | --- | -.14 | 0.056 | .048 |
| Age | .23 | <.001 | .051 | .12 | .008 | .048 | --- | --- | --- | --- | --- | -- |
| Sex*Age | --- | --- | --- | --- | --- | --- | --- | --- | --- | --- | --- | -- |
| Rand. E. | Year & Cap. Zone | | | Year & Cap. Zone | | | Year & Cap. Zone | | | Year | | |
|  | | | | | | | | | | | | |
| Gamma models | | | | | | | | | | | | |
|  | Precise - 100 | | | Broad (5) | | | Broad (10) | | | Broad (14) | | |
|  | Β | p_obt_ | α_sim_ | β | p_obt_ | α_sim_ | β | p_obt_ | α_sim_ | β | p_obt_ | α_sim_ |
| Fixed E |  |  |  |  |  |  |  |  |  |  |  |  |
| # Yrs | .31 | .01 | n/a | .55 | <.001 | n/a | .8 | <.001 | n/a | .69 | <.001 | n/a |
| Sex | -.44 | .008 | .051 | -.05 | .434 | .053 | .01 | .363 | .052 | -.42 | <.001 | .049 |
| Age | --- | --- | --- | .14 | .041 | .05 | .14 | .002 | .05 | --- | --- | --- |
| Sex*Age | --- | --- | --- | --- | --- | --- | --- | --- | --- | --- | --- | --- |
| Rand. E. | Year & Cap. Zone | | | Year & Cap. Zone | | | Year & Cap. Zone | | | Year & Cap. Zone | | |

**Table S3** Mixing matrix by sex showing the proportion of same-sex and opposite-sex connections in two broad scale networks. One network with temporal associations constrained to 10 days (A) and the other with associations constrained to 5 days (B).

|  | Female | Male |
| --- | --- | --- |
| A. Broad scale network (10): |  |  |
| Female | 0.34 | 0.22 |
| Male | 0.22 | 0.22 |
| B. Broad scale network (5): |  |  |
| Female | 0.35 | 0.22 |
| Male | 0.22 | 0.21 |
